# Direct AFM-based nanoscale mapping and tomography of open-circuit voltages for photovoltaics

**DOI:** 10.3762/bjnano.9.171

**Published:** 2018-06-14

**Authors:** Katherine Atamanuk, Justin Luria, Bryan D Huey

**Affiliations:** 1 University of Connecticut, Dept. of Materials Science and Engineering, Storrs, Connecticut 06269, USA

**Keywords:** cadmium telluride (CdTe), photo-conductive AFM (pcAFM), PV performance, solar cell, tomographic AFM

## Abstract

The nanoscale optoelectronic properties of materials can be especially important for polycrystalline photovoltaics including many sensor and solar cell designs. For thin film solar cells such as CdTe, the open-circuit voltage and short-circuit current are especially critical performance indicators, often varying between and even within individual grains. A new method for directly mapping the open-circuit voltage leverages photo-conducting AFM, along with an additional proportional-integral-derivative feedback loop configured to maintain open-circuit conditions while scanning. Alternating with short-circuit current mapping efficiently provides complementary insight into the highly microstructurally sensitive local and ensemble photovoltaic performance. Furthermore, direct open-circuit voltage mapping is compatible with tomographic AFM, which additionally leverages gradual nanoscale milling by the AFM probe essentially for serial sectioning. The two-dimensional and three-dimensional results for CdTe solar cells during in situ illumination reveal local to mesoscale contributions to PV performance based on the order of magnitude variations in photovoltaic properties with distinct grains, at grain boundaries, and for sub-granular planar defects.

## Introduction

Cadmium Telluride (CdTe) is an inexpensive thin-film photovoltaic with ca. 5% of the 2017 global market share for solar cells. To optimize the efficiency and reliability of these, or any electronic devices, a thorough understanding of their composition, microstructure, and performance is necessary as a function of device design, processing, and in-service conditions. Atomic force microscopy (AFM) has been a valuable tool for such characterization, especially of materials properties and device performance at the nanoscale. In the case of thin-film solar cells, local photovoltaic (PV) properties such as the open-circuit voltage, photocurrent, and work function have been demonstrated to vary by an order of magnitude, or more, within tens of nanometers [1–3]. Recently, property mapping with high spatial resolution by AFM has been further combined with the ability to serially mill a surface, in order to reveal underlying surface structures and uniquely develop three-dimensional (3D) nanoscale property maps. The most notable examples are based on pure current detection with the AFM to resolve conduction pathways in filamentary semiconducting devices and interconnects [4–5], and tomographic AFM of photocurrents in polycrystalline solar cells during in situ illumination [6].

Standard photo-conductive AFM (pcAFM) employs a conducting probe, which serves as a positionable top electrode, to map currents upon illumination and/or biasing. With solar cells, the short-circuit current (*I*_SC_) can then be directly visualized by simply measuring the photocurrent when there is no potential difference between the sample and the scanning probe. By further sweeping the bias between the sample and the grounded tip, for a single spot or an array of locations, the resulting *I*–*V* curves can be analyzed to interpret several additional performance metrics, which are widely employed by the PV and solar power communities. The open-circuit voltage (*V*_OC_), for example, is the probe bias necessary for the photocurrent to pass from positive to negative values, i.e. when the solar cell locally transitions from power generation to power shunting. The similarly crucial maximum-power point and/or fill factor can also be identified from *I*–*V* measurements. These and related PV performance parameters are academically, commercially, and more generally societally important given the complexity, functionality, and widespread benefits of PV devices, such as solar cells.

To extract such PV metrics at the nanoscale, for instance with standard 256 × 256 pixel resolution, over 65000 distinct current–voltage spectra must be acquired and analyzed. We previously developed an efficient, method with high spatial resolution for this purpose, namely photo-conductive AFM spectroscopy (pcAFMs) [1], essentially by collecting an entire array of *I*–*V* spectra in parallel via a series of consecutive pcAFM images. Each image is acquired with a sequentially increased sample bias, tracing through the power generation quadrant of the solar cell specimen for a nano- to micro-scale region, all while preserving a measurement location accuracy at the nanometer scale.

However, despite providing spatial resolution as fine as the tip contact area [4], the voltage resolution for pcAFMs clearly depends on the number of voltage steps and range of biases considered. This is a direct function of the number of stable image frames in an area of interest. But a higher voltage fidelity directly equates to a longer overall acquisition time, necessitating both patience as well as imaging and specimen stability that can be a particular challenge for generally fragile materials systems such as molecular perovskites [7]. Traditional point-by-point measurements are far slower still. Consequently, for AFM-based mapping of solar cell performance parameters that are traditionally derived from *I*–*V* measurements_,_ such as *V*_OC_, the spatial and energetic resolution unavoidably conflicts with experimental throughput.

Accordingly, this work presents a new approach for directly mapping *V*_OC_ with nanoscale resolution, requiring a single, standard-speed AFM scan. This leverages the concept of the proportional-integral-derivative (PID) feedback loop that underpins nearly all AFM topography imaging. Normally, this feedback loop continually updates the AFM probe height in order to maintain a constant AFM tip–sample interaction, which is sensed via the integrated cantilever deflection or amplitude that, of course, changes at surface protrusions or depressions. To simultaneously map *V*_OC_ directly, the topography is tracked in the same manner, but a secondary PID loop is also configured to continually adjust the sample bias in order to maintain a photocurrent of zero. This is akin to Kelvin probe microscopy or scanning surface potential microscopy, in which a secondary PID loop varies the sample bias to maintain a fixed cantilever amplitude, phase, or frequency. The capacitive and/or coulombic interactions that perturb these signals null when the probe bias equals the ensemble specimen voltage beneath the tip, providing a directly measured map of local surface properties.

There is a particular need for such efficient direct property-mapping routines for computed tomographic AFM (CT-AFM), in which images are serially acquired during progressive surface milling [6,8]. For instance, to investigate the nearly 50% reduction in efficiency for CdTe solar cells compared to their theoretical limits [9–15], it would be beneficial to have through-thickness *V*_OC_ maps with high spatial and energy resolution. But every *V*_OC_ map, each comprising tens to hundreds of distinct depths through a sample, necessitates tens of consecutive frames via pcAFMs instead of just one image. Equivalent resolution maps from serially acquired individual *I*–*V* measurements are another hundred times slower. Specifically, for relatively standard AFM scanning at a line rate of 0.5 Hz, direct *V*_OC_ imaging as proposed herein requires only ca. 8.5 min (for 256 × 256 pixel resolution), compared to 4.3 h if based on pcAFMs (with 30 voltage steps and, hence, image frames), or 18.2 h for traditional point-by-point studies (based on a duration of 1 s to acquire each spectrum, move to the next location, and settle the probe). Of course, high-speed data acquisition can in principle accelerate such measurements of thousands of discrete spectra, as implemented for “peak force” [16] or “fast force” mapping [17–18] where arrays of force–distance curves are acquired during continuous scanning. However, current detection is generally slower than force transduction due to LRC time constants, and in any case tracing full *I*–*V* curves over a constant range of biases at every location may damage specimens due to occasional high current flow (i.e., heat) or even breakdown. For truly nanoscale tomographic maps of *V*_OC_ and/or *I*_SC_ with minimal specimen damage at tens, hundreds, or thousands of distinct depths throughout a specimen, our substantially faster direct approach presented herein (simply requiring one further image per depth) becomes a practical necessity.

## Results and Discussion

**Figure 1:**
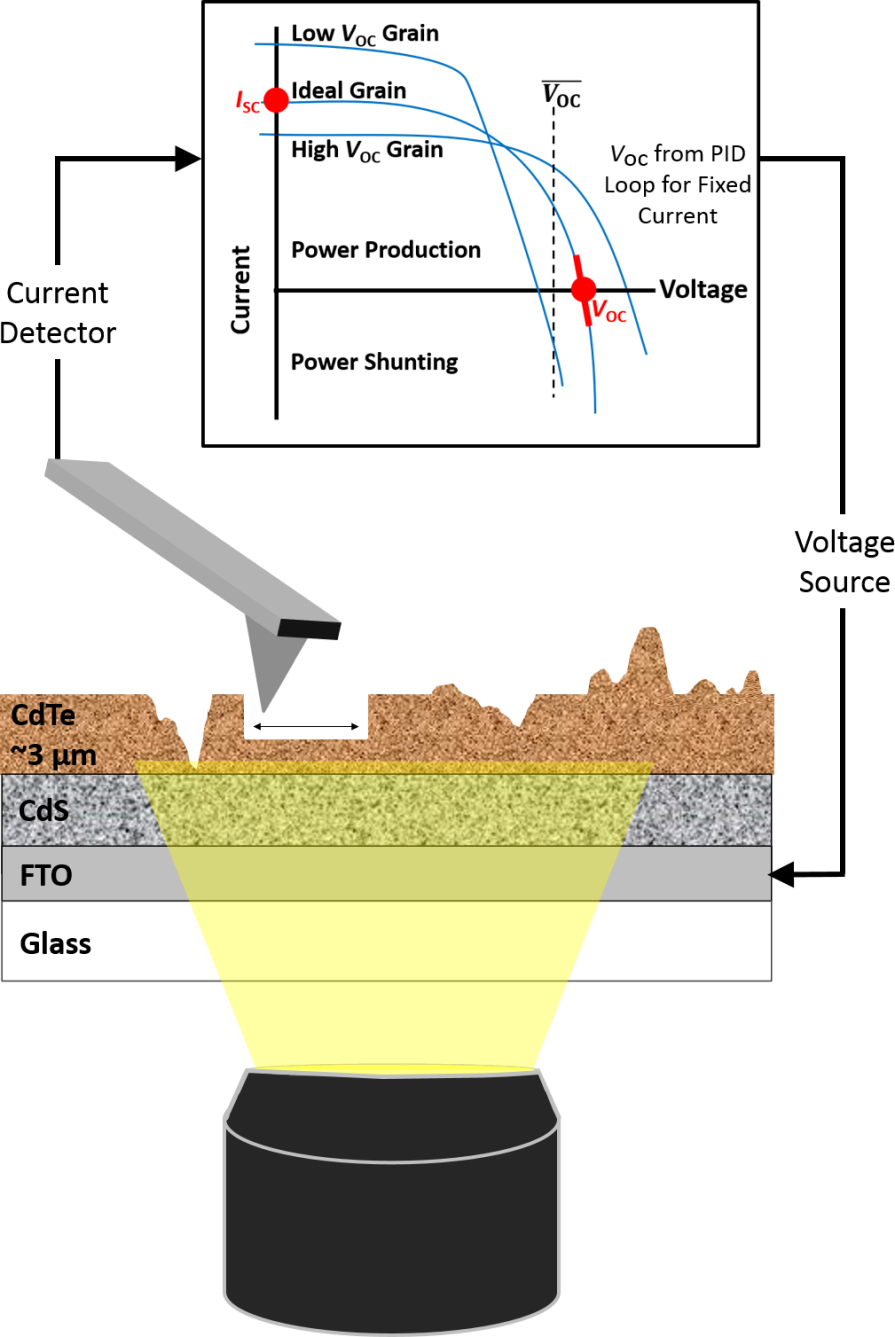
pcAFM measurement of a CdTe/CdS solar cell, during specimen illumination from below through an underlying transparent conducting anode and substrate (FTO/glass). Local currents are detected from above by the AFM probe serving as a positionable cathode. The local photovoltaic properties can vary widely for the heterogeneous microstructure compared to the mean (macroscopic) response. Tomography is achieved by gradually milling the specimen during continuous high-load topographic imaging. Alternating frames toggle between short-circuit current mapping (*I*_SC_) based on the photocurrent at zero bias, and direct open-circuit voltage mapping (*V*_OC_) via a dedicated PID feedback loop continually adjusting the sample bias to maintain a null photocurrent.

Figure 1 depicts the system configuration in such a CT-AFM experiment for *I*_SC_ and direct *V*_OC_ mapping. The AFM (Asylum Research MFP-3D-IO) is custom-mounted on an optical microscope (Nikon TE-2000) that has a broad-spectrum LED light source (Cree MK-R 12). The LED illuminates an area of ca. 1·10^4^ µm^2^, including the sample/tip junction, from below through a 40× objective to provide an intensity of ca. 1 equivalent sun. A conducting (B-doped) diamond-coated silicon AFM probe (Nanoworld CDT-NCHR, Soquel, CA, USA), along with a picoampere-resolution current detector (Asylum Research Orca, model 058, 5 V/nA, 1–10 kHz bandwidth), enable either the short-circuit current to be measured or the open-circuit voltage to be directly determined by engaging the secondary feedback loop as previously described. Since the notations *I*_SC_ and *V*_OC_ are technically defined for 1 equivalent sun, all results and discussions indicate an effective *I*_SC_* and *V*_OC_* because the light source is not a true solar simulator. In any case, lateral spatial resolution remains as fine as ca. 20 nm throughout the measurement according to the final topographic features observed. This is compatible with the tip–sample contact radius for a probe apex with a 25–50 nm radius of curvature and an effectively planar substrate. Tomography is achieved directly with the AFM probe simultaneous to the repeated property mapping. Specific settings include a load of ca. 1 µN, a line rate of 0.5 Hz, and a low-deflection feedback gain producing near “open loop” scanning and hence an essentially planar surface milling [8]. Approximately 15 nm in depth are practically removed per image frame, leading to effective 30 nm resolution in the *z*-direction between consecutive pairs of *I*_SC_* and *V*_OC_* maps throughout the polycrystalline film thickness.

During such progressive imaging some spatial drift is unavoidable, though this is easily accommodated by commercial, free, or custom image analysis routines (respectively Igor Pro, FIJI, and in this case programs written for MATLAB). The necessary drift corrections, typically based on purely rigid registration, cause ca. 10% around the outskirts of the initial property maps to be incomplete for the overall 3D dataset. Accordingly, only pixels with data acquired throughout the depth are considered in the final results. Also, current instead of current density is reported due to the uncertainty about the absolute cross-sectional area.

**Figure 2:**
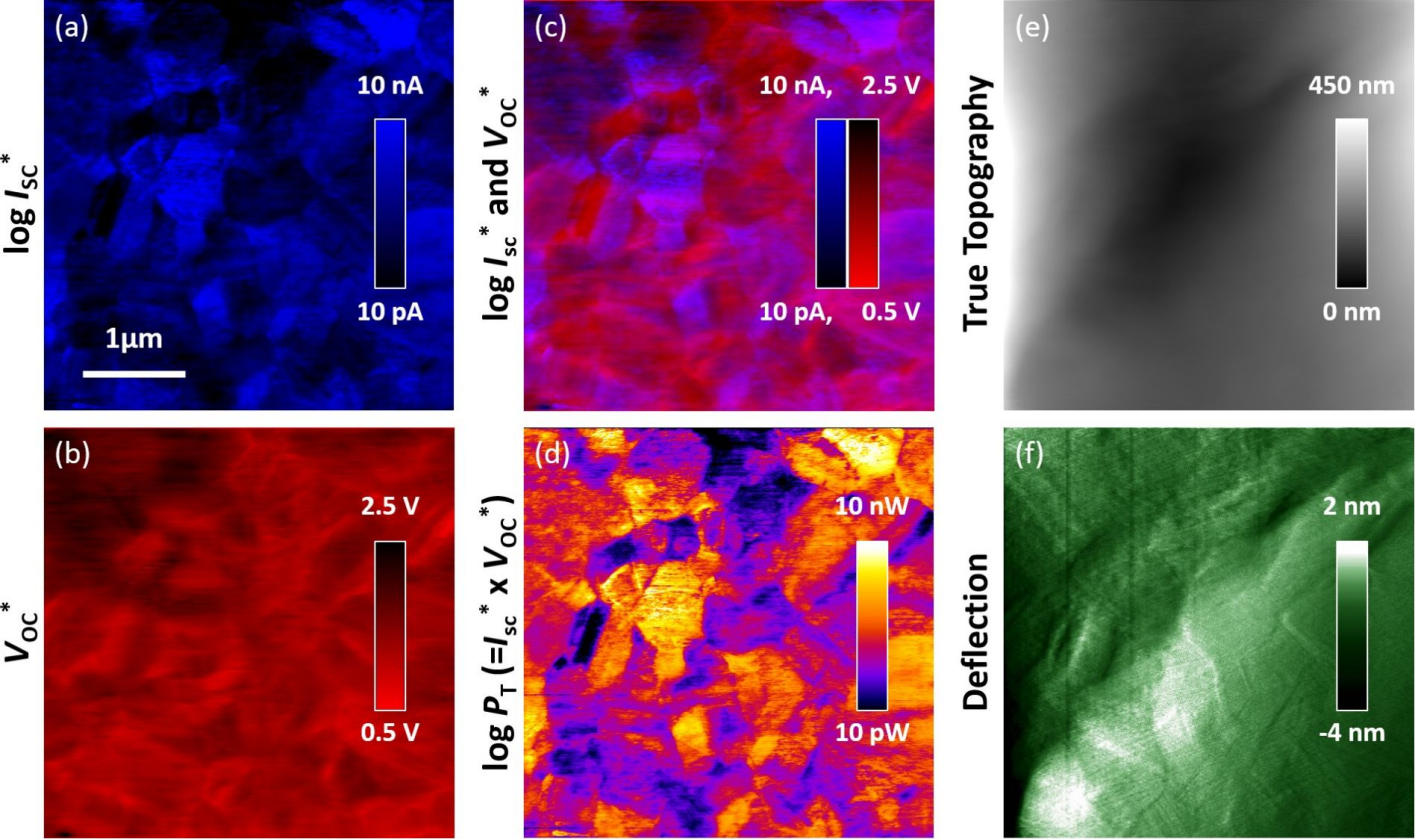
(a) Consecutively acquired *I*_SC_* (note the log scale) and (b) directly measured *V*_OC_* for a single ca. 4 × 4 µm area. (c) A simple overlay of these distinctly colored images is displayed, demonstrating their sometimes complementary and otherwise uncorrelated behavior. The product of *I*_SC_* and *V*_OC_* is shown in (d) indicative of the theoretical power (*P*_T_^*^, log scale). (e) The simultaneously acquired true surface topography and (f) deflection data (essentially an edge-filtered height image to identify any topographic steps or other microstructural boundaries) reveal little correlation with the photovoltaic performance.

Figure 2 presents a representative pair of directly detected, effective short-circuit current (Figure 2a) and open-circuit voltage (Figure 2b) images. These signals are uniquely colored for simultaneous visualization when superimposed (Figure 2c), based on a simple 1:1 overlay of the two distinct color images using conventional image processing software (FIJI). Viewed in this manner, most grains present a consistent *I*_SC_*, which varies up to three orders of magnitude for adjacent grains (note the log scale). *V*_OC_*, on the other hand, is less uniform within a single grain, appearing to vary most strongly at some grain boundaries as well as many seemingly linear features. This type of instant property mapping is especially beneficial for specimens sensitive to their environment, as is common for many photovoltaics in the presence of oxygen or humidity [19]. Along these lines, direct measurements of *V*_OC_^*^ during exposure to acetic acid are ongoing for Si solar cells to correlate any changes in the local properties with this macroscopically known contributor to the accelerated PV degradation in solar panels [20–23]. Here, the short-circuit current and open-circuit voltage signals are further multiplied in Figure 2d to represent the theoretical power. Distinct from the individual or overlain images of Figure 2a–c, this reveals even more spatially localized variations in photovoltaic performance.

As with every study based on scanning probes, it is important to consider any influence of topography on the results. The as-provided surface of the essentially commercial grade polycrystalline film is relatively rough when considered at the nanometer scale, revealing grains, facets, and grain boundaries with topographic protrusions and depressions as great as ±150 nm. Surface-potential studies of a range of photovoltaics have identified correlations between such features and their measured properties, for example with work function differences of molecular perovskites observed at specific facets [24] or grain boundary interfaces [2]. Topography commonly couples with conductive or photoconductive AFM contrast as well. Routines to test for such associations are therefore increasingly employed [1,24], allowing scientists to focus on or ignore such regions depending on whether they are true local variations or experimental artifacts. In any case, in order to minimize the influence of such topography, our specimens are first partially planarized [8]. This provides a surface morphology with slopes gradually transitioning ±5° over several micrometers according to the true topography [25] (Figure 2e). Local protrusions or depressions are smaller than 6 nm per the edge-identifying deflection signal (Figure 2f) acquired simultaneously with Figure 2a. Compared to the as-received surface profile, the RMS roughness is thus improved up to two orders of magnitude. Notably, there are few correlations between this morphology and the photovoltaic performance.

**Figure 3:**
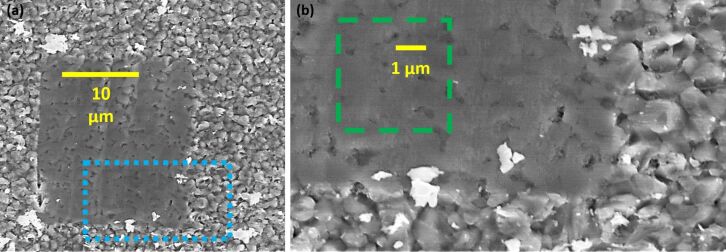
(a) SEM micrograph of a locally planarized polycrystalline CdTe solar cell. The dotted rectangular overlay indicates the location of a higher-magnification SEM image (b) in which the dashed square outline identifies the area studied for Figure 2 and Figure 4.

Figure 3 displays secondary scanning electron microscopy images of the as-provided thin film (Figure 3a, outer regions) and the surface after the initial local planarization (Figure 3a, smooth central area). A corner of this region is depicted in the higher-magnification SEM micrograph (Figure 3b), which also follows ca. 10 nanomilling steps within the square dashed overlay. The sparse bright features around the milled area are clusters of milled material that were not swept out of the field of view during planarization/tomography. The weaker, heterogeneous contrast within results partially from not quite perfect smoothing of the initially high roughness topography.

As already introduced, sequentially repeating *I*_SC_* and direct *V*_OC_* mapping, with sufficient tip force to continuously remove material, leads to a stack of images that uniquely identify local properties in all three dimensions. For simplicity, a constant milling rate of 15 nm/frame is assumed such that each consecutive tomographic slice represents pairs of *I*_SC_* and *V*_OC_* in steps of 30 nm along the *z*-direction. Generally, this assumption is consistent with both the uniform SEM contrast of Figure 3, and the clearly linear features resolved in the images of Figure 2, which simply would not appear to be linear without steady milling. It is more specifically supported by the regularly evolving height from consecutive CT-AFM images in separate measurements through an equivalent 2.2 µm thick planarized film [6]. That study required a nearly uniform number of imaging/milling frames to reveal the clearly identifiable highly conducting back electrode throughout a similar field of view, resulting in an estimated average milling rate error better than ±5 nm per image frame. Of course a more sophisticated 3D interpolation, based on the true (*x*, *y*, *z*) coordinates of each acquired pixel, can be implemented for property maps at even more precise depths or cross sections [26]. Such an approach will be especially warranted for initially rough surfaces or island features, instead of an initially planarized and uniformly milling specimen as studied here.

**Figure 4:**
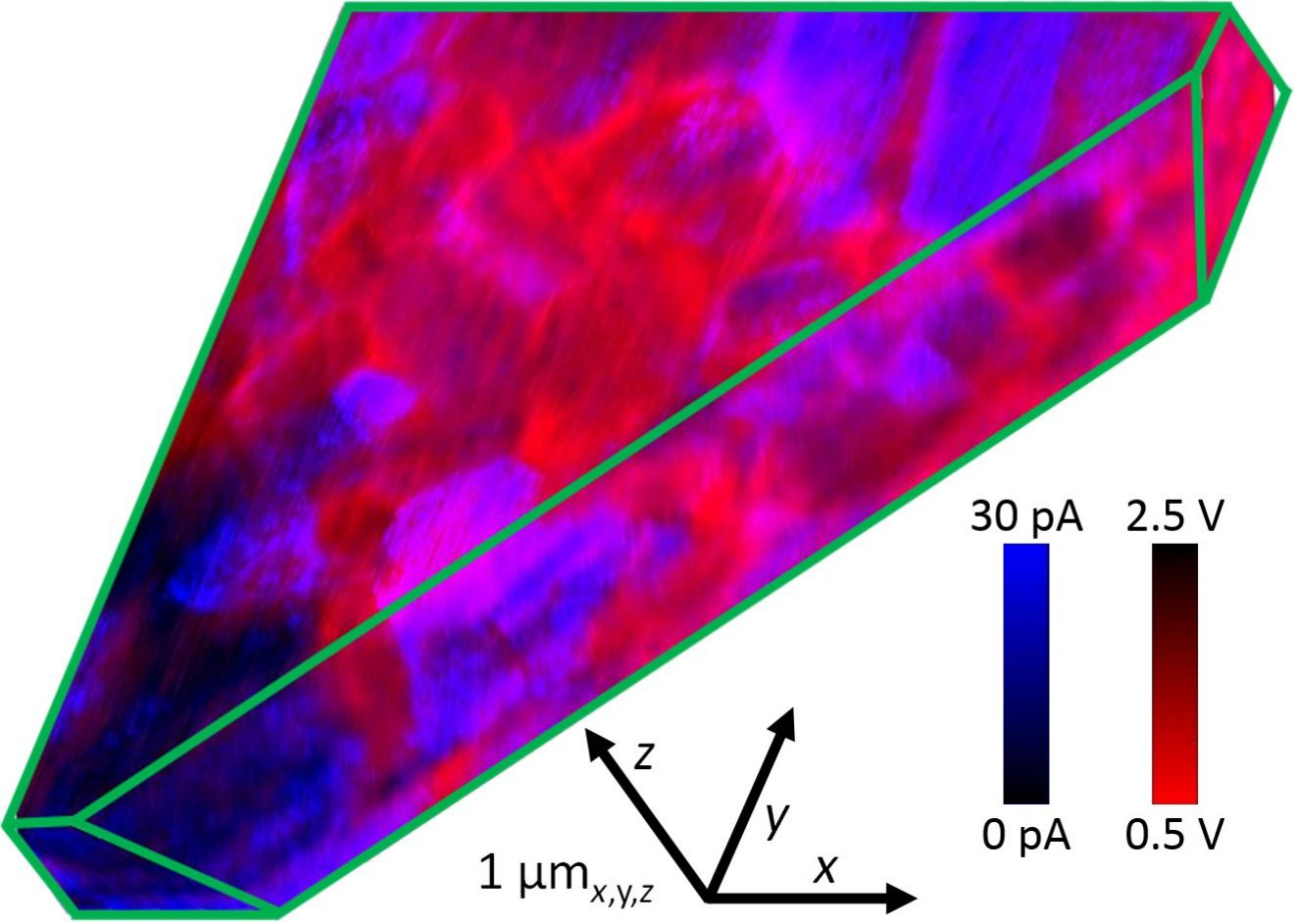
Three-dimensional CT-AFM of the short-circuit current (*I*_SC_*, dark to blue contrast) merged with the directly measured open-circuit voltage (*V*_OC_*, red to dark) volumetrically rendered to partially reveal an *xy* plane, *xz* and *yz* cross sections, and an oblique *xyz* cut to uniquely expose the nanoscale properties through the specimen thickness. The identified axes also serve as scale bars.

The combined *I*_SC_* and *V*_OC_* tomography is 3D-rendered in Figure 4, revealing portions of the full rectangular cuboid of acquired 3D data including: the smooth *xy* planar surface; pure *xz* and *yz* cross sections; and an arbitrary oblique *xyz* section. As with Figure 2, bright contrast identifies areas with a strong *I*_SC_* (blue), while the contrast for *V*_OC_* (red) is flipped to especially highlight the poor *V*_OC_ at grain boundaries and some sub-granular regions. For any given plane through the specimen it is sometimes difficult to recognize these local properties. This is partially due to convolution with inevitable noise in any SPM-based imaging, but especially results from the stacked and arbitrarily shaped and oriented grains in the microstructure of the thin film. When viewed in 3D, however, the directly acquired photovoltaic properties seem clearly correlated with 3D microstructure.

Specifically, the CdTe thin film exhibits profound (orders of magnitude) heterogeneities in local photovoltaic performance within tens of nanometers of crossing a boundary between three-dimensionally adjacent microstructural features. Some grain boundaries as well as sub-granular planar features appear to reveal relatively poor values of *V*_OC_*, supporting prior observations that many interfaces in CdTe may serve as conduits for photoelectrons to move to the underlying n-type CdS layer [6]. Equivalent conclusions have been inferred from complementary techniques such as simple conductive AFM [27], surface-potential mapping [28], and electron beam induced currents [29], though the fully three-dimensional, directly acquired data of *I*_SC_* and now *V*_OC_* in Figure 4 is conclusive.

It is noteworthy that qualitatively similar 3D PV data has been reported based on quasi-*V*_OC_* imaging and tomography, obtained by simply recording the magnitude and sign of currents when biasing at the mean specimen open-circuit voltage [6] or a similarly suitable bias [30]. According to the schematic in Figure 1, positive currents in these cases imply a locally strong *V*_OC_, while negative currents suggest a low *V*_OC_, as exemplified by Figure S1 (Supporting Information File 1). But such quasi-V_OC_^*^ mapping inevitably leads to conjectures based on currents that do not inherently represent actual open circuit conditions, and indeed should be nonlinear near zero current. In fact, non-photovoltaic features such as purely semiconducting, conducting, or resistive regions will appear artificially weak or strong in quasi-*V*_OC_* imaging, as in Supporting Information File 1, Figure S1 and [6] for current-shunting grain boundaries. Figure 2 and Figure 4 reveal a more consistent grain boundary response via the direct measurements. Therefore, although quasi-*V*_OC_* mapping is simple and efficient, spurious contrast mechanisms can mask the actual local *V*_OC_ and corresponding statistical and correlative analyses with microstructure and/or other properties. These can only be best revealed by directly measuring *V*_OC_*, or for even more sophisticated materials property maps by extension of the straightforward approach presented herein. For instance, with appropriate circuitry that multiplies the instantaneously applied bias and the detected photocurrent, the especially important maximum-power point for a solar cell could be directly imaged in 2D or even 3D in future work. This will only require the additional feedback loop constantly adjusting and recording the probe bias to maintain peak power instead of zero photocurrent.

## Conclusion

A new AFM-based method for directly mapping the nanoscale open-circuit potential of photovoltaics is based on a secondary PID feedback loop configured to record local probe biases necessary to constantly maintain open-circuit (zero photocurrent) conditions. In addition to protecting the specimen and probe from high currents as in conventional *I*/*V* sweeps, the efficiency of this single-pass approach for direct *V*_OC_* mapping is beneficial for measurements sensitive to ambient exposure, thermal drift, or multi-image investigations such as tomographic AFM. This is demonstrated in 2D and 3D with CdTe polycrystalline thin-film solar cells, and correlated with effective short-circuit photocurrent mapping. Grain boundaries are directly observed to possess low open-circuit voltages while grain bulks exhibit widely varying short-circuit currents including sub-granular planar features. Variations in these photovoltaic performance metrics are sometimes complementary but also can be uncorrelated, as uniquely observed by overlaying these signals. When considering their product, equivalent to the theoretical power, profound variations are detected at the nano- and micro-scale. Such novel SPM-based measurements can be crucial to advancing the fundamental understanding, and ultimately performance and reliability, of a wide range of photosensors, photoactivated catalysts, and photovoltaics.

## Supporting Information

File 1 Additional experimental data.
