# Supplementary material for: Direct AFM-based nanoscale mapping and tomography of open-circuit voltages for photovoltaics
Source: Beilstein J Nanotechnol. 2018 Jun 14;9:1802–8. doi: 10.3762/bjnano.9.171 (PMC6009312; doi:10.3762/bjnano.9.171)
Supplement: File 1 — Additional experimental data. [file Beilstein_J_Nanotechnol-09-1802-s001.pdf]

Supporting Information

for

Direct AFM-based nanoscale mapping and tomography of open-circuit voltages for photovoltaics

Katherine Atamanuk, Justin Luria and Bryan D. Huey*

Address: University of Connecticut, Dept. of Materials Science and Engineering, Storrs, Connecticut 06269, USA

Email: Bryan D. Huey* - bryan.huey@uconn.edu

* Corresponding author

Additional experimental data

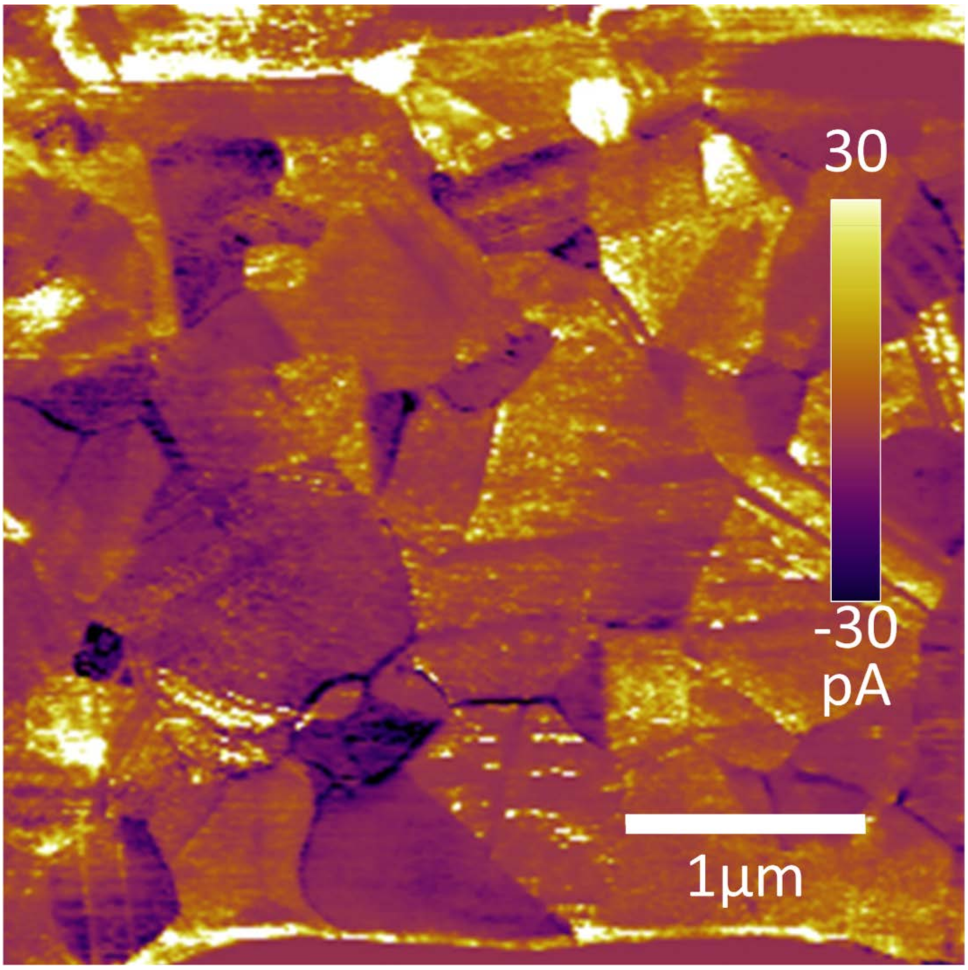

Figure S1: Representative quasi-V_{OC}* image from the measured photocurrent upon illumination during an applied voltage fixed at 700 mV.
